# Common Carotid Artery Volume Flow: A Comparison Study between Ultrasound Vector Flow Imaging and Phase Contrast Magnetic Resonance Imaging

**DOI:** 10.3390/neurolint13030028

**Published:** 2021-06-23

**Authors:** Andreas Hjelm Brandt, Jacob Bjerring Olesen, Ramin Moshavegh, Jørgen Arendt Jensen, Michael Bachmann Nielsen, Kristoffer Lindskov Hansen

**Affiliations:** 1 Department of Diagnostic Radiology, Rigshospitalet, Copenhagen University Hospital, 2100 Copenhagen, Denmark; mbn@dadlnet.dk (M.B.N.); lindskov@gmail.com (K.L.H.); 2 Bk Medical Aps, 2730 Herlev, Denmark; JBOlesen@bkmedical.com (J.B.O.); RMoshavegh@bkmedical.com (R.M.); 3 Center for Fast Ultrasound Imaging, Department of Health Technology, Technical University of Denmark, 2800 Lyngby, Denmark; jaj@elektro.dtu.dk; 4 Department of Clinical Medicine, University of Copenhagen, 2200 Copenhagen, Denmark

**Keywords:** volume flow, vector flow imaging, phase contrast magnetic resonance imaging, common carotid artery

## Abstract

Volume flow estimation in the common carotid artery (CCA) can assess the absolute hemodynamic effect of a carotid stenosis. The aim of this study was to compare a commercial vector flow imaging (VFI) setup against the reference method magnetic resonance phase contrast angiography (MRA) for volume flow estimation in the CCA. Ten healthy volunteers were scanned with VFI and MRA over the CCA. VFI had an improved precision of 19.2% compared to MRA of 31.9% (*p* = 0.061). VFI estimated significantly lower volume flow than MRA (mean difference: 63.2 mL/min, *p =* 0.017), whilst the correlation between VFI and MRA was strong (*R*^2^ = 0.81, *p* < 0.0001). A Bland–Altman plot indicated a systematic bias. After bias correction, the percentage error was reduced from 41.0% to 25.2%. This study indicated that a VFI setup for volume flow estimation is precise and strongly correlated to MRA volume flow estimation, and after correcting for the systematic bias, VFI and MRA become interchangeable.

## 1. Introduction

Appoximately 85% of all strokes are caused by ischemia, while 15% are initiated by hemodynamically significant stenosis of the carotid artery [1,2]. Volume flow quantification of the common carotid artery (CCA) can assess the absolute hemodynamic effect of a carotid stenosis and can be used for monitoring cerebral blood flow after carotid endarterectomy or carotid stenting stenosis [3,4,5].

Phase contrast magnetic resonance imaging angiografi (MRA) and Doppler ultrasound (US) are commonly used for measuring cerebral blood flow [6]. Phase contrast MRA is considered the gold standard for non-invasive cerebral blood flow measurement; however, the estimation of CCA volume flow with MRA is time consuming, the technique is non-mobile, and the evaluation is not performed in real-time [7]. Doppler US is the first choice for carotid flow evaluation, as it is fast, bedside-available, and estimates flow in real-time. While color Doppler is a qualitative method for stenosis assessment in the CCA, spectral Doppler US can provide the CCA volume flow by measuring the mean velocity multiplied with the lumen area found with B-mode imaging. However, spectral Doppler US velocity estimation is angle-dependent and requires manual angle correction [8,9]. Furthermore, Doppler angle correction based on a single angle is insufficient, since in-vivo flow often presents several flow directions [10,11]. Additionally, the spectral broadening effect is ignored, even though spectral broadening causes velocity estimation error at any insonation angle [11,12,13,14]. Several Doppler US studies have investigated the volume flow of carotid arteries; however, evaluation of velocity is still considered the standard method for carotid flow assessment, e.g., for carotid stenosis [1,15,16,17].

Ultrasound vector flow imaging (VFI) is an angle-independent technique for vector velocity estimation [18]. VFI estimates both the axial and the transverse velocity component, while conventional Doppler US only estimates the axial velocity. Given both perpendicular velocities, the vector velocity can be estimated [19]. VFI has been validated with MRA for volume flow estimation in the CCA and with dilution techniques for volume flow in the ascending aorta and arteriovenous fistula [20,21,22,23]. VFI volume flow estimation in the CCA has previously only been conducted in experimental setups with a noncommercial US scanner [20,24].

The aim of this study was to compare the commercial VFI setup for CCA volume flow with the reference MRA method. The hypothesis was that VFI can estimate z similar volume flow as MRA in healthy volunteers.

## 2. Materials and Methods

Ten healthy volunteers (eight males and two females, mean age 32.2 years, range 25–52 years) with no history of cardiac, vascular, or neurologic disease were included after informed consent, with approval obtained from the National Committee on Biomedical Research Ethics (journal no. H-1-2014-FSP-072). Both the left and right CCAs were scanned with VFI in one session. Within the hour before or after the ultrasound examination, MRA recordings of both CCAs of each volunteer for volume flow estimation were obtained. MRA and VFI estimations were performed with the volunteer in the supine position after at least 10 min of rest before each measurement. In a recent paper, MRA and VFI datasets were used for analysis of peak systolic velocities in the CCA [25].

**Figure 1:**
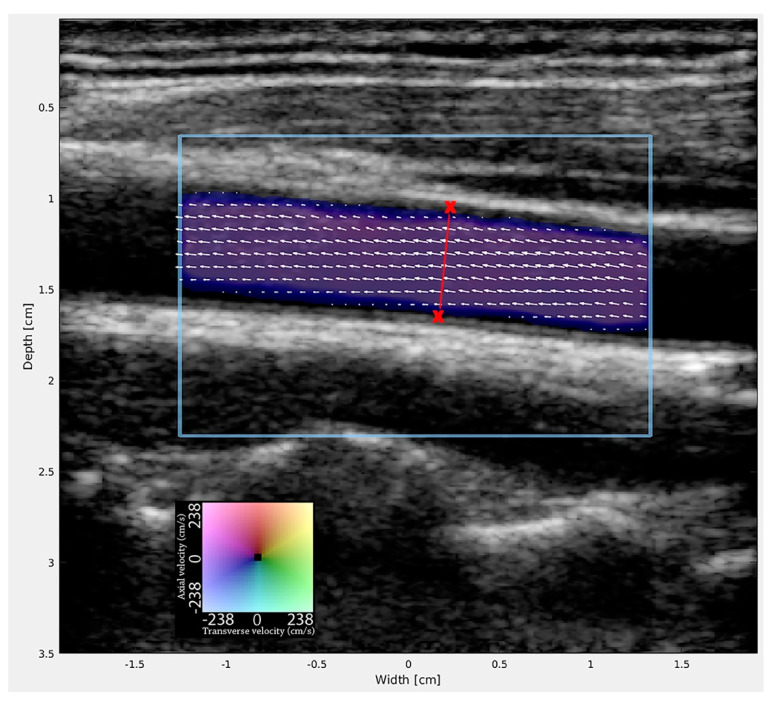
The VFI estimation setup in the CCA. Volume flow was found by integrating the VFI data along a line perpendicular to the long axis of the CCA. The line was drawn between two markers set by the operator at the intima of the vessel. The direction and velocity magnitude of the blood flow are given by the color wheel and indicated by the superimposed vector arrows.

A conventional ultrasound scanner equipped with a VFI (BK3000, BK Medical Aps, Herlev, Denmark) and a linear probe with a frequency range of 8–2 MHz (8L2, BK Medical Aps, Herlev, Denmark) was used to obtain VFI data. VFI was displayed in real-time at 30 Hz in 2D as color-coded pixels superimposed onto the B-mode images. Each color-coded pixel contained quantitative information about the direction and velocity magnitude. Flow was given within a color box adjusted to cover the lumen of the carotid artery with arrows overlaid on the color map to ease the immediate interpretation of the VFI flow data. The pulse repetition frequency was adjusted to the highest velocities to prevent aliasing. To avoid blooming artefacts, wall filter and color gain were set for optimal filling of the vessel by the operator. The transducer was positioned over the longitudinal center line of the vessel in the image plane, where the CCA had the widest diameter with the tunica initma visible at both the superficial and deep vessel walls. VFI recordings were processed offline in MATLAB (Mathworks, Natick, MA, USA) using an in-house developed algorithm for volume flow estimation. The operator placed two markers at the tunica intima on each side of the CCA. Assuming a circular vessel geometry and a circular symmetric flow, the volume flow was estimated along a line drawn between the two points by integration over the entire flow profile of the cross-sectional area, as described previously [26] (Figure 1). The volume flow was given in milliliters per minute and calculated from a minimum of 150 frames corresponding to four to five heartbeats of VFI data. Each CCA was evaluated twice in the same session for precision analyses. Volume flow calculations of the VFI data were performed by a radiologist with five years of VFI experience (A.H.B.) blinded to the corresponding MRA estimates. 

**Figure 2:**
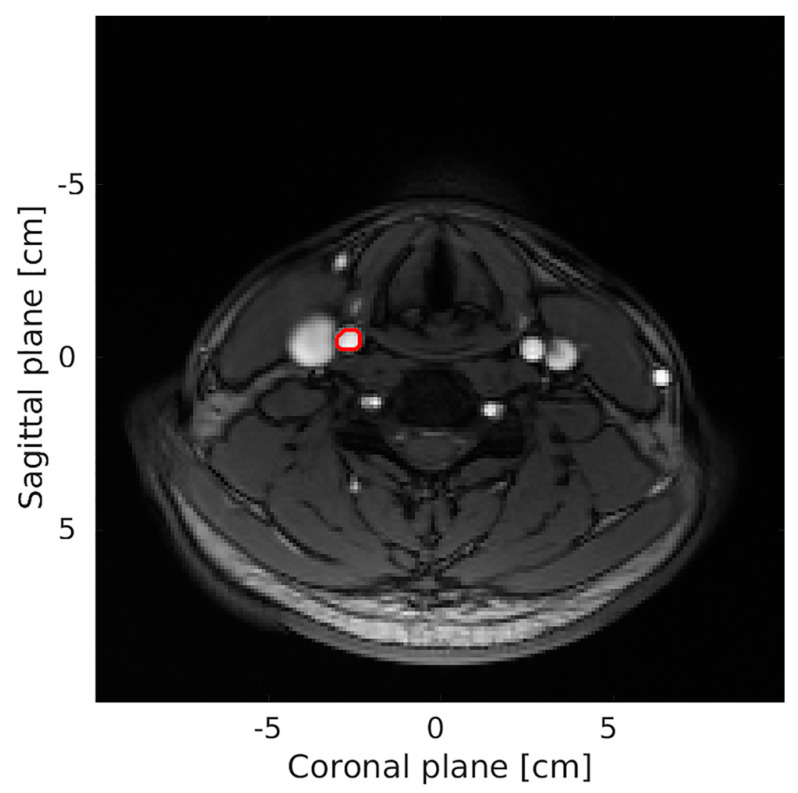
Transverse 2D time-of-flight sequence of the neck obtained with MRA. An example of volume flow of the right CCA was found within the region of interest (**marked red**). The MRA volume flow was found from a corresponding through-plane phase contrast MRA sequence (not shown).

All MRA measurements were performed within 1 h before or after the ultrasound examination. However, one volunteer was scanned 24 h after the VFI scan due to technical issues with the MR scanner. All MRA scans were performed with a 1.5-T MR scanner (Magnetom Avanto, Siemens, Erlangen, Germany). An electrocardiography gated phase contrast sequence using a head and neck coil (Neck Coil, Siemens, Erlangen, Germany) was used to estimate the through-plane velocities of the CCA. The sequence had a repetition time of 42 ms, an echo time of 3 ms, a flip angle 20°, a pixel resolution of 1.1 × 1.1 mm in an image of 216 × 256 pixels, a slice thickness of 5 mm, and a maximum velocity encoding of ±1.0 m/s. For orientation, a 2D time-of-flight sequence with similar resolution was acquired parallel to the applied flow sequence (Figure 2). A radiologist with 15 years of experience (K.L.H.) performed the MRA examination. Each CCA was evaluated twice in the same session for precision analyses; however, due to an error the estimation for the first volunteer, evaluation was only performed once. 

The VFI and MRA volume flow estimations were performed at the same section of the CCA approximately 2 cm downstream from the bifurcation perpendicular to the long axis of the carotid artery. The MRI measurements were processed offline in MATLAB by an in-house developed algorithm. The CCA was pointed out manually and then a boundary detection algorithm, based on the MRA pixel intensity, located the outer contours of the vessel as the region of interest (ROI). All pixels within the ROI were used for the volume flow estimation [27]. All MRA volume flow calculations were performed by J.B.O., blinded to the corresponding VFI estimates.

For the statistical analysis, the precision *P* was found for each method corresponding to two standard deviations (STDs) of the difference between replicate measurements *a* and *b* of a method *x*, divided by the mean, and given as a percentage:

(1) $$P=\frac{2*STD\left(x_{n\;}^{a}-x_{n\;}^{b}\right)}{\bar{x}}\;*100,$$

where *n* is the number of replicated experiments, and $\bar{x}$ is the average of measurements *a* and *b*. 

A linear regression analysis, as well as a Bland–Altman analysis, were used for agreement analyses between VFI and MRA. The first of the two replicated measurements was used for the comparisons. From the limit of agreement (LOA) of the Bland–Altman analysis, the percentage error *PE* was calculated. 

The *PE* for the comparison of two methods *x* and *y* (VFI and MRA) was calculated similarly to the calculation of precision for replicate measurements, i.e., two STDs of the difference divided by the mean of the two methods and given as a percentage:

(2) $$PE=\frac{2*STD\left(x_{n}-y_{n}\right)}{\left(\bar{x}+\bar{y}\right)/2}*100,$$

where *n* is the volunteer number, and $\bar{x}$ and $\bar{y}$ are the average values obtained for methods *x* and *y*. Thus, the precision *P* and *PE* verged toward 0 for perfectly replicated measurements and perfect comparisons. The systematic bias was calculated from the linear regression analysis and applied to VFI data for calculation of a corrected PE, as carried out by Hansen et al. [22].

The expected LOA for the Bland–Altman plot of the two methods *x* and *y* can be calculated as:

(3) $$STD_{x+y}=\sqrt{(STD_{x}^{2}}+STD_{y}^{2}),$$

where *STD* is the standard deviation of methods *x* and *y* in comparison. Instead of *STD*, the calculated precision *P* was used, as per previous research [23,28].

Datasets recorded on the right and left sides were pooled to access a larger amount of data for the comparison. The presence of a statistical difference between the precision of each method, as well as compared to the VFI and MRA estimates, was evaluated with paired *t*-tests. A *p*-value of <0.05 was considered statistically significant. Statistical analyses and data management were performed with Excel (Microsoft Excel (Microsoft Corporation, Redmond, WA, USA), MATLAB, and IBM SPSS Statistics 22 (SPSS Inc., Chicago, IL, USA).

## 3. Results

**Figure 3:**
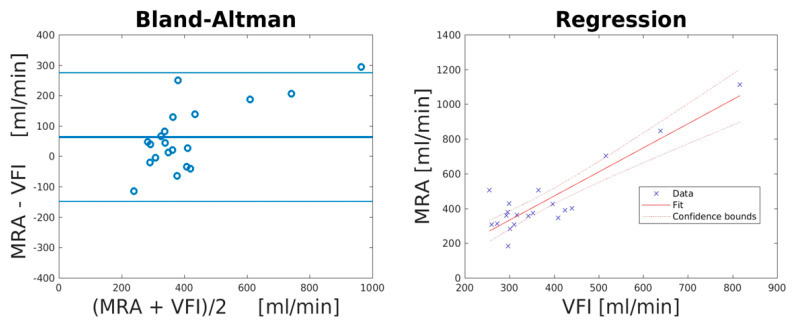
Volume flow estimations with VFI and MRA of the CCA evaluated with Bland–Altman and linear regression plots. The lines in the Bland–Altman plot (**left**) correspond to the mean bias and LOA, while the lines in the linear regression plot (**right**) correspond to line of best fit and confidence bounds. Notice a larger positive bias for higher volume flows, indicating a systematic bias.

An overview of the collected data is given in Table 1. The VFI volume flow was significantly different to the MRA volume flow when comparing the first of the two replicated measurements (*p* = 0.017). The precision estimates (Equation (1)) for VFI and MRA were 19.2% and 31.9% (*p* = 0.061), respectively, which corresponded to an expected LOA using Equation (3) of 37.2%. The mean differences, lower/upper LOA, PE (Equation (2)), and correlation coefficient for the comparisons between VFI and MRA are listed in Table 2 and illustrated in Figure 3. The Bland–Altman plot indicated a systematic bias. After bias correction of the VFI data using the equation for the line of best fit (y = 1.39x − 84.2), the percentage error between VFI and MRA reduced from 41.0% to 25.2%.

## 4. Discussion

This study evaluated the commercial VFI setup against MRA, the reference method for volume flow estimation in the CCA. VFI measured significantly lower volume flow compared to MRA, with indication of an improved precision. The correlation between MRA and VFI was strong. To test if the two methods are interchangeable, the LOA of a Bland–Altman plot in a comparison study should not be wider than the expected LOA, or below 30% as recommended by Critchley et al. [29]. The PE was above the excepted LOA and 30%, and therefore, VFI and MRA cannot be considered interchangeable. However, the Bland–Altman plot indicated a systematic bias. After bias correction, the reduced PE was below 30%, as well as the expected LOA, suggesting that the two methods are interchangeable if the VFI data are corrected. Hence, this study indicates that a commercially available VFI setup for volume flow estimation after bias correction is interchangeable with the reference method MRA and may be ready for volume flow evaluation in a clinical context.

Blood flow volume measurement can be estimated with conventional Doppler US; however, conflicting results have been published concerning the accuracy of both in vitro and in vivo estimations. In vitro experiments have shown a reasonable accurate measurements of blood volume flow in the flow range of 100–1000 mL/min with an average root mean square error of 37.2–81.1 mL/min between a flow phantom and several Doppler ultrasound systems, while in vivo experiments of the carotid arteries have shown Doppler US volume flow estimation to overestimate 22–46% compared to MRA [4,30].

VFI volume flow estimation with a non-commercial setup has, in previous studies, shown a strong correlation (*R =* 0.91) with MRA [20]. Volume flow estimation with a commercially available VFI setup as the one applied in this study has previously been investigated with a different algorithm for volume flow calculation in arteriovenous fistulas and the ascending aorta [22,31]. The algorithm for volume flow estimation used in these studies assumed a circular symmetric and parabolic flow profile, while the algorithm used in this study only relied on circular symmetric flow. The obtained VFI volume flow estimations presented in the previous studies were not interchangeable with the compared methods, i.e., thermodilution and Doppler US [22,31].

In diseased vessels with stenotic segments, the velocity increases and the volume flow decreases [17,32]. While, the intima media thickness in the common carotid artery has been used as a surrogate marker for atherosclerosis [33], the preferred method for stenosis assessment using ultrasound is velocity evaluation within the stenotic vessel segment [34]. However, due to calcified plaques, the degree of stenosis from velocity assessments can be difficult to estimate in some patients, and volume flow estimation with VFI downstream of the stenosis could be an alternative measure [35]. 

The complexity of the flow also increases with stenoses, and can be assessed with spectral Doppler US by estimating spectral broadening or evaluating mosaic patterns with color Doppler [36,37]. An alternative to conventional Doppler for the assessment of flow complexity is VFI, whose angle can independently, instantaneously, and quantitatively estimate whether the flow is laminar or turbulent [38]. In both the CCA and the superficial femoral artery, the flow changes induced by stenoses have been quantified with VFI and compared to digital subtraction angiography with a strong correlation [39,40]. VFI may improve stenosis grading by estimation of volume flow and assessment of flow complexity. All CCAs examined in this study had laminar flow. The VFI volume flow estimation algorithm applied in this study must therefore be examined on vessels with disturbed flow to explore the VFI method for patients with vessel disease.

VFI is not limited by the size or placement of the sample volume as with Doppler US. The placement of the sample volume and the manual applied angle correction in Doppler US evaluation add to the error in the velocity estimation and can be a reason for the low interobserver agreement found for conventional Doppler US [41,42,43,44]. VFI may be less operator dependent than conventional Doppler US, since no manual angle correction is applied [45]. Recently, VFI has been shown to have improved inter- and intraobserver agreement and to be less operator experience dependent compared to Doppler US [46]. Doppler US has previously been compared to MRA and has shown to estimate significantly different volume flow estimates [30]. Meanwhile, the commercial VFI setup has shown to be more precise than Doppler US for peak systolic velocity evaluation in the CCA, a study comparing the commercial VFI setup for volume flow estimation against Doppler US is missing [25].

Quantitative assessment of carotid and vertebral volume flow has been suggested as an estimate of total cerebral blood flow and can be clinically useful in several of cerebrovascular diseases. Besides evaluating carotid stenosis, the presence of collateral pathways and associated conditions, investigation of vertebrobasilar insufficiency, and estimation of the shunt volume in cerebral arteriovenous malformations have been suggested [7]. An accurate measurement technique is therefore of great importance. VFI might be more accurate than MRA for volume flow estimation, even though MRA is accepted as the gold standard for quantification of cerebral blood flow [47]. Therefore, VFI may be a new tool for total cerebral blood flow evaluation. 

Some drawbacks of this study must be acknowledged. The small sample size is the most obvious limitation. Furthermore, the applied algorithm did not concern movement of the vessel during the cardiac cycle or displacement of the transducer during the scan. The lack of tracing the vessel wall may have caused an underestimation of the velocity profile. Moreover, volume flow estimation was not performed directly on the scanner, but rather processed offline. A fully automatic integrated method for volume flow assessment could ease the pursuit for a larger, more evidence-creating study. Only one operator performed the data collection and data analysis for VFI; hence, no interobserver variability was calculated. Furthermore, even though MRI is considered the gold standard for non-invasive volume flow estimation, the method underestimates the flow by 5–8% with a standard deviation of 11.2% for repeated measurements, which obviously could affect the comparison analyses [47]. Finally, alignment between MRA and VFI recordings was not carried out exactly, which will bias the comparison of velocities [48]. 

## 5. Conclusions

In healthy volunteers, VFI and MRA volume flow estimates of the CCA were strongly correlated, though VFI significantly underestimated the volume flow estimates. VFI had superior precision compared to MRA and was, after correction of the systematic bias, interchangeable with MRA. VFI may be a useful alternative for volume flow estimation in the CCA with bedside availability and a lower operational cost compared to MRA.

## Figures and Tables

**Table 1:** Mean and range of the replicated MRA and VFI estimations. (1) represents the first acquisition and (2) the second.

	MRA (1) (*n* = 20)	MRA (2) (*n* = 18)	VFI (1) (*n* = 20)	VFI (2) (*n* = 20)
**Mean (mL/min)**	447.0	421.3	380.3	385.6
**Range (mL/min)**	182.5–1111.4	280.6–696.8	260.4–816.6	235.2–753.6

**Table 2:** Mean differences, lower/upper limits of agreements (LOAs), percentage errors (PEs), and correlation coefficients for comparisons between VFI and MRA.

	Mean Difference (mL/min)	Lower LOA (mL/min)	Upper LOA (mL/min)	Correlation Coefficient (*R*^2^)	PE (%)	PE after Correction of Systematic Bias (%)
**MRA vs. VFI**	63.2 (95% CI: −113.4 to 12.9)	−148.8 (95% CI: −235.9 to 61.7)	275.1 (95% CI: 188.0–362.2)	0.81 (*p* < 0.0001)	41.0	25.2

## Data Availability

The data presented in this study are available on request from the corresponding author. The data are not publicly available due to privacy.
